# Paraspinal muscle characteristics on MRI in degenerative lumbar spine with normal bone density, osteopenia and osteoporosis: a case-control study

**DOI:** 10.1186/s12891-022-05036-y

**Published:** 2022-01-20

**Authors:** Gengyu Han, Da Zou, Zexiang Liu, Siyu Zhou, Wei Li, Chunjie Gong, Zhuoran Sun, Weishi Li

**Affiliations:** 1 Department of Orthopaedics, Peking University Third Hospital, No. 49 North Garden Road, Haidian District, Beijing, 100191 China; 2 Engineering Research Center of Bone and Joint Precision Medicine, Ministry of Education, Beijing, China; 3 Beijing Key Laboratory of Spinal Disease Research, Beijing, China

**Keywords:** Bone mineral density, Hounsfield units value, Magnetic resonance imaging, Osteoporosis, Paraspinal muscle

## Abstract

**Background:**

To investigate the difference of paraspinal muscles in patients with normal bone density, osteopenia and osteoporosis.

**Methods:**

Patients undergoing surgery for lumbar spinal stenosis were included. Thirty-eight patients with osteoporosis were matched to patients with osteopenia and patients with normal bone density in a 1:1 manner according to WHO criteria. Dual-energy X-ray absorptiometry (DXA) scans and lumbar CT were performed preoperatively to measure the BMD of lumbar, femur and hip and HU values of L1-L4 respectively. The relative total cross-sectional area (rTCSA) and fat infiltration (FI) of multifidus (MF) and erector spinae (ES), and the relative functional CSA (rFCSA) of psoas major (PS) were measured at L4–5 and L5-S level on preoperative MRI.

**Results:**

Osteoporotic patients showed lower BMI, higher MF FI and higher ES FI when compared with normal bone density group (25.57 ± 3.71 vs 27.46 ± 3.11; 0.38 ± 0.1 vs 0.32 ± 0.08; 0.33 ± 0.1 vs 0.28 ± 0.08; all adjusted *p* < 0.05). Both the MF FI and ES FI were significantly correlated with lumbar T-score (*r* = − 0.223, *p* < 0.05; *r* = − 0.208, *p* < 0.05) and the averaged lumbar HU value (*r* = − 0.305, *p* < 0.01; *r* = − 0.239, *p* < 0.05).

**Conclusions:**

Osteoporosis and paraspinal muscle degeneration might interact with each other and coexist in patients with degenerative lumbar diseases. It is recommended that the paraspinal muscle degeneration should be considered simultaneously when finding a patient with low bone mass before surgery.

## Background

Osteoporosis characterized by low bone mineral density (BMD) is an increasingly major public health issue in aging societies [1]. Commonly, the geriatric patients who need spine surgery have osteoporosis meanwhile [2]. The osteoporosis may adversely influence the surgical outcomes in patients with lumbar degenerative diseases, such as increasing the risk of proximal junctional kyphosis and screw loosening [3, 4].

Paraspinal muscle is important for spinal segmental stability and the maintenance of spinal alignment [5, 6]. Paraspinal musculature is also influenced by physiological processes like aging [7]. It is widely accepted that paraspinal muscle degeneration is correlated to multiple degenerative diseases [8]. Studies have demonstrated that paraspinal muscle degeneration might also lead to several complications after lumbar surgery [9, 10]. Considering the muscle and bone are interconnected musculoskeletal units, assessing the association of paraspinal muscle characteristics with the vertebral column is of increasing value.

Some studies have investigated the relationship between paraspinal muscle characteristics based on magnetic resonance imaging (MRI) and bone quality [11–13]. However, inconsistent results were reported by Sollmann et al. that the FI assessed by muscle attenuation was not correlated to the BMD [13]. Besides, there is no research on the possible correlation between osteoporosis and paraspinal muscle degeneration in degenerative lumbar spine requiring fusion surgery.

Against this background, we aimed to compare the difference of paraspinal muscles by quantitative MRI measurement in degenerative lumbar spine requiring surgery with normal bone density, osteopenia and osteoporosis matched by age and sex. Considering the limitation of dual-energy X-ray absorptiometry (DXA) in degenerative lumbar spine, we also used the vertebral Hounsfield units (HU) value based on computed tomography (CT) to evaluate the BMD. Therefore, we also elucidated the correlation between paraspinal muscle degeneration and bone density measured by CT HU.

## Methods

This retrospective study was approved by the Institutional Review Board, with the requirement for informed consent being waived. We reviewed hospitalized patients undergoing posterior lumbar fusion for lumbar spinal stenosis between July 2015 and December 2015. We diagnosed lumbar spinal stenosis through a combination of clinical history, physical examination and radiological changes showing spinal canal stenosis on MRI. Inclusion criteria included (1) aged ≥ more than 45 years, (2) underwent lumbar MRI, lumbar CT and DXA of lumbar, femur and hip before surgery. Exclusion criteria were (1) previous spinal surgery, (2) patients with bone tumor, ankylosing spondylitis, diffuse idiopathic skeletal hyperostosis, rheumatoid arthritis, tuberculosis, or secondary osteoporosis, (3) previous or current hormone therapy. A total of 334 patients were identified.

To identify the difference of paraspinal muscles in patients with normal bone density, osteopenia and osteoporosis, we selected the normal bone density group and osteopenia group from the fusion patients who were matched in a 1:1 manner to the osteoporosis patients according to age (the difference was less than 3 years) and sex. As a result, 114 patients were selected in this study (38 patients for each group, 63 females and 54 males, mean age 60.32 ± 6.02 years, BMI 26.55 ± 3.71 kg/m^2^). Of them, 107 patients were lumbar spinal stenosis and 7 patients had spinal stenosis with spondylolithesis (grade I). Table 1 shows baseline characteristics of study population.

**Table 1:** The baseline characteristics of study population

Variables	Total (*n* = 114)
Gender (male/female)	54/60
Age (yr)	60.32 ± 6.02
Body mass index (kg/m2)	26.55 ± 3.71
Diagnosis of patients (number)	
Spinal stenosis	107
Spinal stenosis + Spondylolithesis	7

### BMD evaluation

**Fig. 1:**
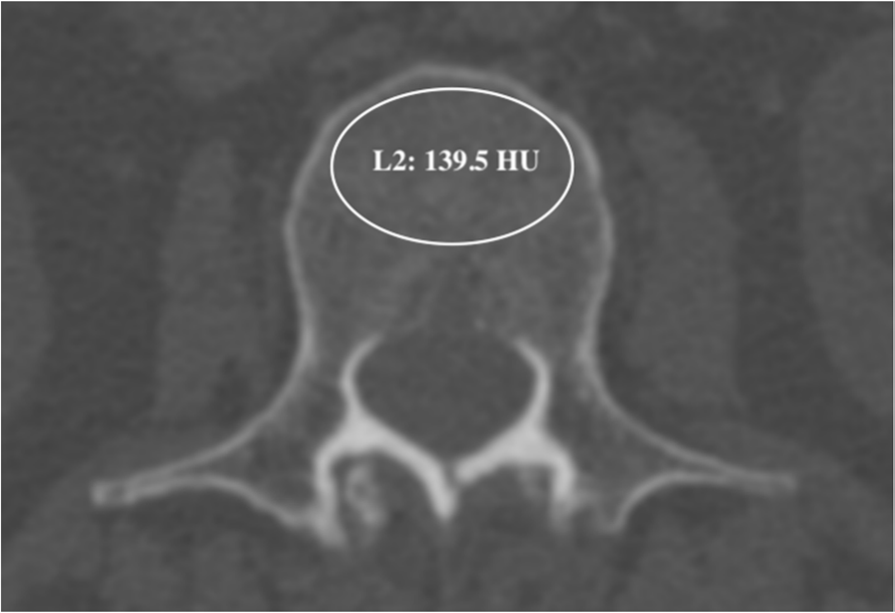
Example of the measurement of HU value at L2 (a 58-year-old woman): the HU value was 139.5

For BMD, DXA scans (Discovery A densitometers, Hologic Inc., Bedford, MA, USA) of lumbar, femur and hip and three-dimensional reconstructive lumbar CT (Siemens, DEFINITION, tube voltage 120 kV) were performed preoperatively. The BMD and *T*-score of L1-L4, femoral neck and hip were recorded from DXA. The HU values of L1-L4 were measured for each patient according to the method of previous studies (Fig. 1) [14]. An oval region of interest inclusive of trabecular bone was placed in the middle-axial CT image of vertebral body. The cortical bone and posterior venous plexus were excluded in the measurement. We utilized the WHO criteria to distinguish osteoporosis (*T*-score ≤ − 2.5) from osteopenia (− 2.5 < *T*-score < − 1) and normal BMD (*T*-score ≥ − 1) for all included patients. The lowest *T*-score was chosen for diagnosis.

### Paraspinal muscle evaluation on MRI

**Fig. 2:**
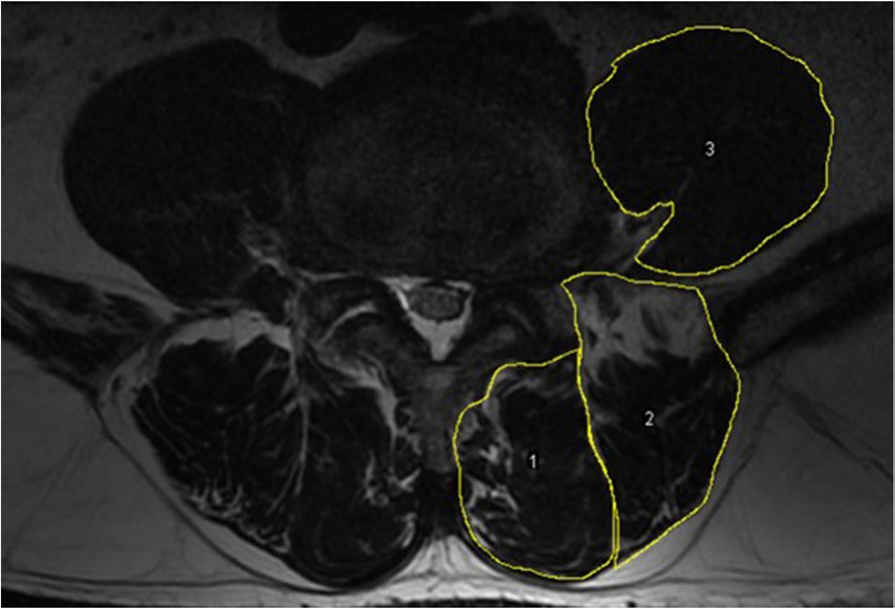
Measurements of paraspinal muscular parameters on axial T2-weighted MRI (a 63-year-old woman). Regions of total cross-sectional area of multifidus (1), erector spinae (2) at L4–5 level were outlined by yellow lines. For psoas muscle, only functional muscle was outlined by yellow lines

**Fig. 3:**
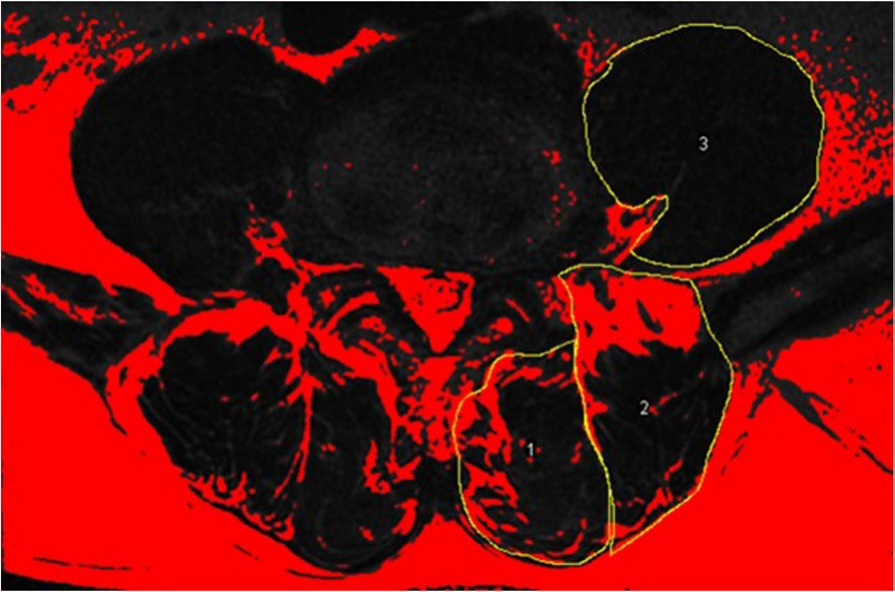
Thresholding technique to highlight intramuscular fat area (red area)

All enrolled patients had undergone preoperative MRI of lumbar area with Signa HDxt 3.0 T (General Electric Company). The axial MRI was parallel to the inferior endplate of the vertebral body and the slice thickness was 3 mm with a 3-mm gap between each slice. We measured the multifidus (MF), erector spinae (ES) and psoas major (PS) bilaterally from T2-weighted images at the center of the intervertebral disc of L4–5 and L5-S level. The following parameters were measured on each level by the Image J software (Fig. 2): total cross-sectional area (TCSA, including muscle, intramuscular fat and soft tissue) of MF, ES and intervertebral disc; FI of MF and ES was measured by the previously reported thresholding technique (Fig. 3) [15, 16]; For PS, only functional cross-sectional area (FCSA) was measured in view of the outline of intramuscular fat and soft tissue was not clearly defined (Fig. 2) [10]. We calculated the mean value of cross-sectional area (CSA) and FI of L4–5 and L5-S to reflect the whole muscle profiles in lower lumbar [11, 16]. Relative cross-sectional area (rCSA, the ratio of cross-sectional area of muscle to that of disc at the same level) was introduced for reducing the effect of body shape on muscular parameters [10, 17]. rCSA of both total muscle (T) and functional muscle (F) were measured as rTCSA and rFCSA.

To test the reliability, all muscular parameters of 15 patients were randomly selected and were measured by two observers independently. After 3 weeks, the same measurements were performed by one observer.

### Statistical analysis

The age- and sex-matching process was performed with the case-control matching function of SPSS. Statistical difference analysis of clinical characteristics and paraspinal muscle parameters between the three groups (normal bone density, osteopenia, and osteoporosis) were performed using Kruskal-Wallis H test, and *P* values were adjusted using Bonferroni correction. Chi-square test was also used for categorical data. Partial correlation analyses were used to analyze the relationship between T-scores, HU values and the paraspinal muscle parameters with the control of BMI. Intraclass correlation coefficient (ICC) of the intra- and inter-reader reliability for muscle parameters was calculated (two-way random, absolute agreement, and single measures). Statistical significance was set at *P* value < 0.05. All statistical analyses were performed using SPSS 22.0 (IBM Corp).

## Results

The clinical characteristics of the participants and quantitative measurements of paraspinal muscles are summarized in Table 2. After matching the sex and age among the three groups, the osteoporotic patients presented with lower BMI, higher MF FI and higher ES FI when compared with normal bone density group (25.57 ± 3.71 vs 27.46 ± 3.11; 0.38 ± 0.1 vs 0.32 ± 0.08; 0.33 ± 0.1 vs 0.28 ± 0.08; all adjusted *p* < 0.05). Whereas no significant difference in BMI, the MF FI and ES FI was found between normal bone density group and osteopenia group (all adjusted *p* > 0.05), and between osteopenia group and osteoporosis group (all adjusted *p* > 0.05). Besides, there were no statistically significant differences in MF rTCSA, ES rTCSA and PS rFCSA among three groups (all *p* > 0.05).

**Table 2:** The comparison of clinical characteristics and paraspinal muscle characteristics among the normal bone density, osteopenia and osteoporosis group

	Normal bone density	Osteopenia	Osteoporosis	*P* value
Gender (male/female)	18/20	18/20	18/20	1
Age (years)	60.16 ± 6.37	60.24 ± 6.07	60.55 ± 5.78	0.997
BMI (kg/m2)	27.46 ± 3.11†	26.61 ± 4.08	25.57 ± 3.71†	0.02
With spondylolithesis	2	1	4	0.499
*T*-score of lumbar (g/cm2) ‡	1.17 ± 0.87	−0.48 ± 1.29	−2.36 ± 0.7	< 0.001
*T*-score of femoral neck (g/cm2) ‡	−0.23 ± 0.63	−1.63 ± 0.43	−2.17 ± 0.66	< 0.001
*T*-score of hip (g/cm2) ‡	0.22 ± 0.62	−1.05 ± 0.54	−1.69 ± 0.57	< 0.001
minimum *T*-score‡	−0.39 ± 0.54	−1.89 ± 0.35	−3.19 ± 0.48	< 0.001
HU value of L1-L4‡	157.76 ± 29.1	124.27 ± 27.71	95.23 ± 26.39	< 0.001
L4 + 5				
MF rTCSA	0.53 ± 0.11	0.48 ± 0.14	0.51 ± 0.12	0.088
ES rTCSA	0.65 ± 0.14	0.65 ± 0.17	0.64 ± 0.19	0.85
MF FI	0.32 ± 0.08†	0.34 ± 0.07	0.38 ± 0.1†	0.032
ES FI	0.28 ± 0.08†	0.32 ± 0.16	0.33 ± 0.1†	0.042
PS rFCSA	0.64 ± 0.13	0.6 ± 0.16	0.61 ± 0.13	0.306

*Abbreviations*: *BMI* Body mass index, *ES* Erector spinae, *FI* Fat infiltration, *HU* Hounsfield units, *MF* Multifidus, *PS* Psoas major, *rFCSA* Relative functional cross-sectional area, *rTCSA* Relative total cross-sectional area

“†” represented two groups had a significant difference (adjusted *p* < 0.05), “‡” represented three groups had a significant difference with each other respectively (all adjusted *p* < 0.05)

Correlation analysis showed that the averaged MF FI of L45 and L5S level had a significant correlation with lumbar T-score, femoral neck T-score, hip T-score and minimum T-score when controlling for BMI (*r* = − 0.228, *p* < 0.05; *r* = − 0.213, *p* < 0.05; *r* = − 0.192, *p* < 0.05; *r* = − 0.242, *p* < 0.01; Table 3). The averaged ES FI of L45 and L5S level had a significant correlation with lumbar T-score and minimum T-score (*r* = − 0.208, *p* < 0.05; *r* = − 0.218, *p* < 0.05; Table 3). Besides, there was a positive correlation between the averaged PS rFCSA and T-score of femoral neck (*r* = 0.227, *p* < 0.05; Table 3). Both MF rTCSA and ES rTCSA had no correlation with any T-scores (all *p* > 0.05; Table 3).

**Table 3:** The relationship between bone mineral density and paraspinal muscle characteristics controlling for BMI tested by linear regression

L4 + 5	*T*-score of lumbar	*T*-score of femoral neck	*T*-score of hip	Minimum *T*-score
MF rTCSA	0.141	0.176	0.157	0.123
ES rTCSA	0.066	0.124	0.132	0.057
MF FI	−0.223*	−0.224*	−0.192*	−0.25**
ES FI	−0.208*	− 0.137	− 0.134	− 0.218*
PS rFCSA	0.146	0.227*	0.167	0.158

*Abbreviations*: *ES* Erector spinae, *FI* Fat infiltration, *MF* Multifidus, *PS* Psoas major, *rFCSA* Relative functional cross-sectional area, *rTCSA* Relative total cross-sectional area

*p < 0.05, **p < 0.01

Both the averaged MF FI and ES FI of L45 and L5S level were correlated with the HU values at each level and the averaged HU value of L1-L4 (For MF FI, *r* = − 0.291; *r* = − 0.334; *r* = − -0.283; *r* = − 0.262; *r* = − 0.305; all *p* < 0.01; For ES FI, *r* = − 0.243; *r* = − 0.23; *r* = − 0.215; *r* = − 0.228; *r* = − 0.239; all *p* < 0.05; Table 4). Moreover, the averaged PS rFCSA of L45 and L5S level was also correlated with the HU values at L2 level and L4 level and the averaged HU value of L1-L4 (*r* = 0.195; *r* = 0.206; *r* = 0.196; all *p* < 0.05; Table 4).

**Table 4:** The relationship between HU value and paraspinal muscle characteristics controlling for BMI tested by linear regression

L4 + 5	L1HU	L2HU	L3HU	L4HU	Average HU of L1-L4
MF rTCSA	0.184	0.178	0.196*	0.217*	0.202*
ES rTCSA	0.168	0.196*	0.203*	0.118	0.178
MF FI	−0.291**	−0.334**	− 0.283**	−0.262**	− 0.305**
ES FI	−0.243*	− 0.23*	−0.215*	− 0.228*	−0.239*
PS rFCSA	0.181	0.195*	0.168	0.206*	0.196*

*Abbreviations*: *ES* Erector spinae, *FI* Fat infiltration, *HU* Hounsfield units, *MF* Multifidus, *PS* Psoas major, *rFCSA* Relative functional cross-sectional area, *rTCSA* Relative total cross-sectional area

*p < 0.05, **p < 0.01

The ICCs for both intra-rater and inter-rater reliability of MF rTCSA, ES rTCSA, MF FI, ES FI and PS rFCSA were all > 0.8 (Table 5).

**Table 5:** Intra-rater and inter-rater reliability of paraspinal muscle parameters using intraclass correlation coefficient

L4 + 5	Intra-rater	Inter-rater
MF rTCSA	0.948	0.915
ES rTCSA	0.917	0.896
MF FI	0.897	0.823
ES FI	0.933	0.815
PS rFCSA	0.867	0.845

*Abbreviations*: *ES* Erector spinae, *FI* Fat infiltration, *MF* Multifidus, *PS* Psoas major, *rFCSA* Relative functional cross-sectional area, *rTCSA* Relative total cross-sectional area

## Discussion

We found that BMI of subjects with normal bone density was significantly higher than that of subjects with osteoporosis. The result was consistent with previous studies [18, 19]. As BMI is an indicator of whole-body fat, the patients with large BMI might have a higher production of estrogens generated by adipose tissue and a large gravitational effect caused by increased body weight, thus obtaining a large bone mass.

FI has been considered a crucial component of paraspinal muscle degeneration. Our study demonstrated that osteoporotic patients showed higher MF FI and higher ES FI at L4-S level when compared with normal bone density group. Kim et al. reported that the FI of paraspinal muscles in patients with osteoporotic spinal compression fracture was higher than those without fractures [20]. Besides, Zhao et al. also found that FI of MF, ES and PS of subjects with normal bone density were all significantly less than those with osteopenia and those with osteoporosis, and there was an inverse correlation between paraspinal muscle FI and BMD [12]. These agrees with our finding that both the MF FI and ES FI were significantly correlated with lumbar T-score and minimum T-score. It is known that muscle contraction force can be applied into predicting BMD at various locations [21]. The mechanism involved might be poor muscle strength and function caused by high FI in paraspinal muscles, which could reduce the mechanical loading on bone [22]. Consequently, the increase of intramuscular fat deposition in lumbar spine could be an important indicator for low BMD. In consequence, we recommended that surgeons should conduct a BMD evaluation before surgery when finding a high FI in paraspinal muscles, as osteoporosis is a risk factor for complication.

Of note, increased intramuscular fat in lumbar spine might have an inverse effect on BMD when compared with whole body fat according to our findings. A study demonstrated a low negative correlation between paraspinal muscle density and BMI [23]. They deemed that body fat probably did not settle in the last two lumbar levels. Thus we considered the increased FI of paraspinal muscles was a risk factor for poor bone quality, while the incremental BMI was a protective factor.

Our analysis showed no statistical difference between osteoporosis, osteopenia and normal groups in terms of paraspinal muscle CSAs. Lee et al. revealed the similar results that no statistical difference between osteoporotic and non-osteoporotic groups in lumbar extensor muscles and PS area [11]. Our findings might also support Abbas et al’s study that paraspinal muscle CSAs correlated with spine instability rather than bone quality as all groups have lumbar spinal stenosis [24]. We also found that MF rTCSA and ES rTCSA had no correlation with any T-scores. In Lee et al.’s study, lumbar BMD showed statistically significant correlation with paraspinal muscles CSA. And Sollmann et al. found a significant correlation of BMD and the CSA ratio (PS CSA divided by ES CSA) [13]. A possible interpretation might be that the relationship between CSA and muscle strength was not as significant as that of FI [22]. Thus, the decrease of CSA may not lead to a declining mechanical loading on bone. Another reason might be the different measurement and parameters in two studies. Though no statistically significant difference in PS rFCSA among three groups, there was a positive correlation between it and T-score of femoral neck. A study demonstrated that smaller PS CSA was significant correlated with decreased relative flexion strength [22]. Considering the structure of the PS terminates at the lessor trochanter of the femur, a small PS FCSA could generate a low mechanical loading on bone.

Moreover, many studies have also recommended the vertebral Hounsfield units (HU) value based on computed tomography (CT) can evaluate the BMD with excellent reliability and good performance in diagnosis in recent years [25, 26]. Whether the HU value of lumbar spine is relevant to the paraspinal muscle atrophy and FI on MRI is still indistinct. Interestingly, we found that both CSA and FI of paraspinal muscles were more relevant to the HU value of lumbar by CT than BMD by DXA. Previous study has reported that decreased muscle area on CT occurred 5.7 times more frequently in cases of reduced bone density measured by HU value [27]. This indicated that a patient with low bone density detected by CT preoperatively was more inclined to have a severe paraspinal muscle degeneration than by DXA in the meantime. Based on this finding, surgeons should do some precautionary measures like more rigid fixation or more graft bone to reduce the risk of complications if they find a patient with poor bone quality on preoperative CT, since the patient may be accompanied by severe paraspinal muscle degeneration.

We recognized limitations in the present study. First, we focused on the patients who needed to undergo surgery for lumbar spinal stenosis, thus the results might not be generalized to community people. Besides, several included patients combined with low-grade spondylolisthesis which might be a confounder. Moreover, we could not exclude the negative impact of spinal pathology on muscles. It is possible that symptomatic lumbar diseases have aggravated back muscle degeneration in the first place. However, our results indicated that osteoporosis and paraspinal muscle degeneration had a correlation and coexisted in inpatients with degenerative lumbar diseases. It is recommended that the surgeons should also pay close attention to the paraspinal muscle degeneration during making a surgical decision.

## Conclusion

In patients with lumbar fusion for lumbar degenerative diseases, osteoporotic group showed lower BMI, higher MF FI and higher ES FI at L4-S level when compared with normal bone density group. In linear regression, we found the MF FI and ES FI were significantly correlated with both lumbar T-score and the averaged HU value of L1-L4. PS rFCSA were also positive correlated to the averaged HU value of L1-L4. Paraspinal muscle morphology had a stronger correlation with lumbar BMD measured by CT than by DXA. It is recommended that the paraspinal muscle degeneration should be considered simultaneously when finding a patient with low bone mass before surgery, thus the surgeons can prepare some precautionary measures to reduce the risk of complications.

## Data Availability

The datasets used and/or analyzed during the current study are available from the corresponding author on reasonable request.

## Abbreviations

BMI: Body mass index
CT: Computed tomography
CSA: Cross-sectional area
DXA: Dual-energy X-ray absorptiometry
ES: Erector spinae
FCSA: Functional cross-sectional area
FI: Fat infiltration
HU: Hounsfield units
ICC: Intraclass correlation coefficient
MF: Multifidus
MRI: Magnetic resonance imaging
PS: Psoas major
rFCSA: Relative functional cross-sectional area
rTCSA: Relative total cross-sectional area
TCSA: Total cross-sectional area
